# Weight loss and modeled cost savings in a digital diabetes prevention program

**DOI:** 10.1002/osp4.665

**Published:** 2023-03-07

**Authors:** OraLee H. Branch, Mohit Rikhy, Lisa A. Auster‐Gussman, Kimberly G. Lockwood, Sarah A. Graham

**Affiliations:** ^1^ Lark Health Mountain View California USA

**Keywords:** DPP, medical cost savings, obesity, type 2 diabetes

## Abstract

**Background:**

Participation in the National Diabetes Prevention Program (DPP) can improve individual health through reduced risk of type 2 diabetes and save the healthcare system substantial medical costs associated with a diagnosis of type 2 diabetes and its associated complications. There is less evidence of outcomes and cost savings associated with a fully digital delivery of the DPP.

**Methods:**

This study assessed 13,593 members who provided an initial digital weight and subsequently achieved various weight loss and engagement outcomes during their participation in a digital DPP. Analyzed data included both complete observations and missing observations imputed using maximum likelihood estimation. Findings include members' behavioral correlates of weight loss and a literature‐based cost‐savings estimate associated with achieving three mutually exclusive weight loss or engagement benchmarks: ≥5% weight loss, >2% but <5% weight loss, and completion of ≥4 educational lessons.

**Results:**

11,976 members (88%) provided a weight after 2 months of participation, enabling calculation of their weight nadir. Considering complete data, 97% of members maintained or lost weight. Using the imputed data for these calculations, 32.0% of members achieved ≥5%, 32.4% achieved >2% but <5%, 32.0% maintained ±2%, and 3.6% gained weight. Members who lost the most weight achieved their weight nadir furthest into the program (mean day = 189, SE = 1.4) and had the longest active engagement (mean days = 268, SE = 1.4), particularly compared to members who gained weight (mean nadir day = 119, SE = 3.7; active engagement mean days = 199, SE = 4.9) (both *p* ≤ 0.0001). Modeled 1‐year cost‐savings estimates ranged from $11,229,160 to $12,960,875.

**Conclusions:**

Members of a fully digital DPP achieved clinical and engagement outcomes during their participation in the program that confer important health benefits and cost savings.

## Graphical abstract

Members of a commercially available, fully digital Diabetes Prevention Program achieved weight loss and engagement outcomes during their participation in the program that confer important health benefits and cost savings. A conservative, literature‐based cost‐savings model demonstrated the potential financial value associated with achieving weight loss and engagement outcomes in a digital DPP.

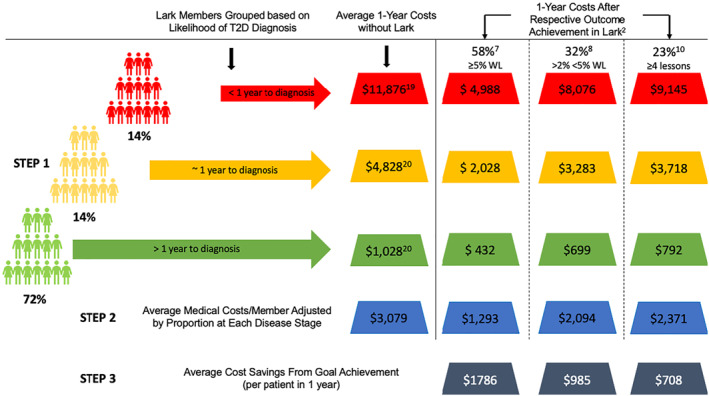

## 1 INTRODUCTION

Three‐quarters of the US population is either overweight or obese,^1^ and the typical annual weight gain of US adults is approximately 0.34–0.71 kg.^2^, ^3^, ^4^ Losing weight is of critical importance for individuals who are overweight or obese because of the relationship between body weight and risk for type 2 diabetes (T2D)^5^ and the strong evidence that weight loss can delay the progression from prediabetes to T2D.^6^, ^7^, ^8^ Lifestyle modification programs that can facilitate weight loss are recommended for all individuals who are overweight or obese.^5^, ^9^ Research has shown that individuals at high risk for diabetes who are not enrolled in a lifestyle modification program like the National Diabetes Prevention Program (DPP) gain ∼0.42 kg per year.^10^ Increasing enrollment in the DPP is an ongoing challenge, but digital health offerings improve the availability of accessible programs by meeting participants when and where they need assistance.^11^, ^12^, ^13^ Digital solutions can also improve the personalization of coaching interactions through the large volume of data they collect from members and the frequent feedback delivery and reminders made possible via automation.^14^

Participation in a digital DPP can improve both individual health through disease prevention and save the healthcare system substantial medical costs by reducing the incidence of T2D and its associated complications. Although some enrolled participants lose a substantial amount of weight in these programs, there is variability in the magnitude of weight loss.^15^ Studying the participants who lose the most weight helps to elucidate behavioral correlates of achieving weight loss.

Clinically meaningful weight loss is often defined as ≥5% of initial body weight.^16^ There are direct physiological benefits associated with losing ≥5% including improved glucose, insulin, and triglyceride profiles.^17^ Attaining ≥5% weight loss reduces the risk of T2D by 58%.^7^, ^18^ Beyond the direct health benefits for the patient, this reduction in diabetes risk is also cost saving for payers because it reduces the excess medical costs associated with a diagnosis of T2D.^19^, ^20^, ^21^

Using claims data, Craff et al.,^19^ found that a diagnosis of T2D was associated with an excess of $11,876 in medical costs in the year of diagnosis. Similarly, Visaria et al.,^22^ compared the mean annualized all‐cause costs of patients with incident T2D versus matched controls and found that the mean cost after diagnosis was $13,221 higher for those with T2D. Khan et al.,^20^ estimated that individuals who escalated from prediabetes to T2D had an additional medical cost of $4828 in the year including the diagnosis. This agrees with Visaria et al.,^22^ who found that individuals 1 year before T2D had mean all‐cause costs $6563 greater than a matched sample that did not convert to T2D. There are also creeping costs occurring years before a T2D diagnosis. Khan et al.,^20^ observed that costs rose during the 5 years leading up to a new incident T2D diagnosis.

Achieving ≥5% weight loss is not the only outcome of a lifestyle modification program that confers benefits. For individuals losing 2%–5%, physiological benefits include glycemic improvements and reduced systolic blood pressure and triglycerides.^17^ As Ryan et al.,^17^ asserted, “patients need not reach a BMI <25 kg/m^2^ in all instances, but can be healthier at any weight, as long as it is a reduced weight.” Multiple studies have shown that small improvements in body weight are beneficial for lowering diabetes risk.^8^, ^17^ Hamman et al.,^8^ estimated a 16% reduction in the risk of converting to T2D for every 1 kg of weight loss. There is even evidence that maintaining weight (i.e., preventing weight gain) reduces the risk of a T2D diagnosis.^10^, ^23^

Beyond the direct health benefits and cost savings of weight loss and maintenance, there are also benefits to engaging in the educational curriculum of a DPP. Campione et al.,^10^ observed that members of a lifestyle modification program who completed ≥4 educational lessons, which educate on healthy lifestyle behaviors such as diet and physical activity, had a 46% reduction in risk of T2D even in the absence of weight loss. This reduction in T2D may be attributed to educational and motivational content focusing on healthy lifestyle concepts other than weight loss and the subsequent increase in preventive care and decrease in unnecessary hospitalizations that may occur as a result.^24^ Taken together, the benefits of lifestyle modification programs are attractive to individuals who want to improve their overall health and reduce their risk for T2D.

Although interested individuals can self‐pay to participate in a lifestyle modification program like the DPP, the important health benefits and potential for healthcare cost savings provide support for the DPP being a covered health service. For the payers who provide coverage for these programs, it is important to understand the proportion of individuals who achieve various health‐related outcomes in these programs and the associated potential for cost savings.

The purpose of this study was to assess the prevalence of weight loss among overweight and obese adults from data gathered by a covered health service, fully digital DPP called Lark that is powered by conversational artificial intelligence (AI). The program has full CDC recognition (organization # 4358176) and is delivered via a mobile application where members can communicate 24/7 with the AI coach via a text message‐like conversational interface. Results of this study include the proportion of members who achieved various weight loss and engagement outcomes and their behavioral correlates and estimated cost savings associated with achieving healthy lifestyle outcomes. The primary hypothesis was that members who engaged to a greater extent and remained in the program for a longer period would achieve larger amounts of weight loss resulting in greater cost savings.

## 2 METHODS

### 2.1 Study design

This was a retrospective, observational study of members of the Lark DPP who enrolled between 1 January 2021, and 7 February 2022, provided an initial digital weight, and completed at least one educational lesson demonstrating minimal intent to engage in the program. Study participants agreed to Lark's privacy policy, which included permission to use their de‐identified data for research. The study purpose was to identify the proportion of members who achieved various health outcomes in a fully digital DPP, identify the behavioral correlates of successful weight loss, and demonstrate the potential for cost savings based on outcome achievement.

### 2.2 Participants and recruitment

The Lark DPP is a covered health service that members may choose to participate in under their health insurance plans or employers. Eligible members who opted into the program received a link via text message to download the program to their iOS or Android smartphones. Inclusion criteria for this study were the CDC requirements for DPP participation^25^: (1) over 18 years old; (2) no previous diagnosis of type 1 or 2 diabetes; (3) initial BMI ≥25 kg/m^2^; and those who met at least one of the following items 4 or 5: (4) blood test result (within past year) of either fasting plasma glucose between 100 and 125 mg/dl, 2‐hour plasma glucose after 75‐g glucose load between 140 and 199 mg/dl, or hemoglobin A1c 5.7%–6.4%; or 5) a risk score indicating high risk for T2D.^26^ Thus, individuals need not have an official diagnosis of prediabetes to participate, but they must be at high risk for T2D.

### 2.3 Description of the program

The Lark DPP is a fully digital lifestyle modification program described in detail elsewhere.^27^ Briefly, the program is deployed on an iOS or Android smartphone and is powered by conversational AI. This means that participants engage synchronously with the AI “coach” on demand via a text‐based interface. The experience is much like texting on a smartphone. The AI coach provides healthy lifestyle coaching; gives feedback on behaviors such as logging physical activity, a meal, or sleep; offers encouragement; and is available to converse 24/7. The accessibility of an AI coach is key to offering “just‐in‐time” support for participants when they are interested in engaging.

The educational content of the National DPP PreventT2 curriculum^28^ is also delivered to participants via this text‐based interface. The AI coach provides participants with the approved DPP curriculum of 26 lessons, each delivered over the course of a week. A participant must complete seven lesson “check‐ins” (no more than one per day) to complete one lesson. The curriculum is spaced per CDC requirements delivering the first 16 “core” lessons focused on weight loss over the first 6 months and the final 10 “maintenance” lessons spread over the remaining 6 months.

### 2.4 Calculation of weight loss

Program members weigh themselves using connected digital scales. Weights are automatically transmitted to the digital platform and tracked longitudinally. For this study, the primary weight‐loss outcome was the weight nadir that occurred any time after 2 months in the program. Members were weight “Gainers” if they gained >2% of their initial body weight, “Maintainers” if they remained weight stable between ±2%, “Partial Achievers” if they lost >2% but <5%, and “Achievers” if they lost ≥5%. The authors also present the average number of members within each weight‐loss category based on their monthly nadir (rather than only a single peak) to provide a conservative range for the proportion of members achieving the above outcomes.

Once a member confirms their initial weight, Lark tracks their weight trajectory for the duration of the program. Outlier detection algorithms ensure fidelity of all collected weight data. The algorithms automatically discard neighboring weights that represent greater than a ±7 lb deviation from the previous weight. Additionally, during post processing, an algorithm flags weight measurements that do not follow a reasonable weight‐loss trajectory based on a member's initial weight. To identify unlikely weight‐loss trajectories, the algorithm applies thresholds of what a reasonable amount of weight loss would be over time. To create these thresholds of reasonable weight loss, the algorithm only considers members with a valid first and last weight. A valid first weight means that all weights provided within the first 10 days of a member's activity in the program did not deviate from each other by more than 5%. Similarly, all weights provided in the 10 days leading up to a final weight could not deviate by more than 5%. With these constraints in place, the algorithm calculates the average and SD of weight loss for the entire group over different time windows during the program (0–60, 60–90, 90–180, 180–270, and 270 + days). The use of time windows accounts for the rate of weight changing over time (i.e., greater near program start, tapering off over time). The algorithm removes weight measurements for any member who had a weight that fell outside of ±2 SD of the average during the respective time window.

### 2.5 Behavioral correlates of weight loss

A fully digital DPP can leverage member data to determine which behaviors are associated with achieving various amounts of weight loss. Member demographics collected as a part of program participation include age, sex, race, and ethnicity. Member characteristics include initial weight and BMI, diagnosis of prediabetes, physical activity level at baseline, any self‐reported diagnosis of T2D during the program, and Patient Health Questionnaire (PHQ‐2) score as a depression screener.^29^ The remaining data demonstrate the ways in which members interact with program components and features of the digital platform. These engagement data include educational lessons completed, interactions with the AI coach, weekly physical activity, meals logged, weigh‐ins, and total days with any form of active program engagement within the app.

### 2.6 Cost‐savings model

Estimated cost savings associated with weight loss in this program are based on literature detailing the risk of converting to T2D, costs associated with a diagnosis of T2D, and the reduction in risk of being diagnosed with T2D if members achieved various outcomes during their participation in the DPP. The excess costs associated with a diagnosis of T2D came from two sources: The first source was costs reported in a study by Milliman^19^ that matched Lark DPP members to individuals contained in a large database of medical claims. The database, Milliman MedInsight Emerging Experience, is a research database of nationwide de‐identified healthcare claims data for individuals from 2017 to 2021, as described in.^19^ The second source included the costs described in Khan et al.,^20^ that represent the costs for the time before T2D since the authors designed the paper to consider the costs that creep up in the years before T2D, and because this paper provided a lower, more conservative estimate than shown in the baseline (pre‐year) of Visaria et al.^22^ The risk stratification of converting to T2D and the above‐described costs can be viewed in Table 1.

**TABLE 1:** Literature‐based model of estimated conversion rate to type 2 diabetes (T2D) and next‐year excess costs associated with being at risk for T2D diagnosis.

Risk category	% of program members likely to convert to T2D	Next‐year average costs of converting to T2D	Description of cost estimates
High risk (diagnosis imminent <1 year)	14%	$11,876	Excess costs at T2D conversion and the year after (subtracting pre‐T2D diagnosis costs).^19^ Members in this study expected to imminently convert to T2D classified as high risk.
Medium risk (diagnosis ~1 year away)	14%	$4828	Excess costs in the year closest to T2D diagnosis. Khan et al.,^20^ modeled the increasing medical costs years before a T2D diagnosis. The year before T2D conversion, they observed an average excess cost of $4828. This cost estimate agrees with the excess pre‐T2D costs described in Visaria et al.,.^22^ Members in this study at this stage of disease classified as medium risk of T2D in the following year.
Low risk (diagnosis >1 year away)	72%	$1028	Average excess costs 2–5 years before T2D diagnosis estimated using Khan et al.,^20^ study years 2010‐2013. The average of these 4 years (representing years 2‐5 before T2D conversion) was $1028. Rising costs of prediabetes also observed by Craff et al.,^19^ with an even higher excess cost following a new conversion to prediabetes. Members in this study at this stage of disease classified as low risk of T2D in the following year.
Weighted average cost per member in following year if NOT enrolled in a DPP		$3079	

Approximately 14% of individuals convert to T2D annually.^7^, ^30^ The incident T2D rate is currently increasing with even asymptomatic COVID‐19 leading to an increased chance of conversion to T2D.^31^ To approximate the risk of conversion to T2D for members in the present study, members fell into one of three hypothetical groups: high risk for those predicted to imminently convert (14% of members), medium risk for those predicted to convert in the near future should they not be in a year‐long DPP (14% of members), and low risk for whom conversion is not in the near‐term future but their medical costs are increasing in the years prior to T2D (72% of members) (Table 1). The low‐risk group includes everyone not considered to be high or medium risk to make the model clear and represent individuals for whom conversion is not in the near‐term future, but medical costs are rising.

Regarding a reduced risk of T2D conversion, members received an assignment to one of three levels of risk reduction based on their observed weight loss. For members who achieved ≥5% weight loss, the landmark DPP study observed a 58% reduction in the risk of T2D.^7^ Members who achieved >2% but <5% received a 16% reduction in the risk of T2D diagnosis for every 1 kg weight loss based on the findings of Hamman et al.,.^8^ To simplify this calculation for modeling purposes, all members in this category received a 32% risk reduction for losing at least 2 kg (∼2% weight loss for a person starting at 220 lbs). Finally, the remaining members who did not reach one of these weight‐loss achievements received a risk reduction only if they completed ≥4 educational lessons of the DPP. A recent study demonstrated that regardless of weight change or loss, individuals who completed ≥4 DPP lessons had a 46% reduction in T2D diagnosis.^10^ It is likely that some of these individuals lost weight, and some gained (resulting in an overall nonsignificant change in BMI); thus, instead of the full 46% risk reduction, members only received a 23% risk reduction for completing ≥4 lessons. This decision reflects cutting 46% in half, assuming a normal distribution of weight change, to remove the benefits likely associated with the members who lost weight. Of note, 23% is very close to the 20% reduction in T2D risk observed by Feldman et al.,^23^ if overweight or obese adults were to maintain their weight in middle age. Similarly, Milliman^19^ showed that the T2D progression rates for people with prediabetes who participated in a DPP (had completed ≥1 session) were 3‐ to 10‐fold lower than those who did not participate in a DPP. An explanation for the reduction in risk associated merely with DPP participation may be that cost savings are also related to factors other than diabetes diagnosis—prior research has demonstrated that there are cost savings associated with diabetes self‐management education (DSME), specifically due to fewer hospitalizations for those who complete a DSME program and greater adherence to preventive care visits.^24^

### 2.7 Statistical analyses

The authors conducted all statistical analyses in R Studio version 4.0.5. The descriptive analyses characterize the demographics and characteristics of the overall sample and assess differences across weight loss subgroups. The primary analyses considered the number of members who Gained weight (>2%), Maintained weight (±2%), Partially Achieved weight loss (>2% but <5%), and Achieved weight loss (≥5%), and the average weight loss and timing of peak weight loss for each of these groups. Weight results include both complete data and imputed weight and nadir day data for the 1617 members who were missing weight data after 2 months. Missing data treatment involved imputation using expectation maximization via the Amelia package in R^32^ with appropriately specified bounds for each variable. Variables included in the imputation model are those displayed in Table 1, as well as coaching conversations as an indicator of engagement in the program during the active weeks. The resulting imputed data reflect the pooled averages and SDs across five imputation iterations. Analyses of the behavioral correlates of each weight‐loss category included ANOVAs for comparing the groups on continuous data and chi‐squared tests for categorical data.

To demonstrate potential cost savings, the authors applied the cost‐savings projections to the number of members achieving each respective outcome (≥5% weight loss, >2% but <5% weight loss, and completion of ≥4 lessons) and summed the total. To provide a range for the cost‐savings estimate, the authors additionally considered the average percentage of members in each achievement category per month (rather than at a single point in time at the weight nadir) and summed these totals.

### 2.8 IRB approval

This study received exemption status from Advarra Institutional Review Board (#Pro00047181) for retrospective analyses of previously collected and deidentified data.

## 3 RESULTS

**FIGURE 1:**
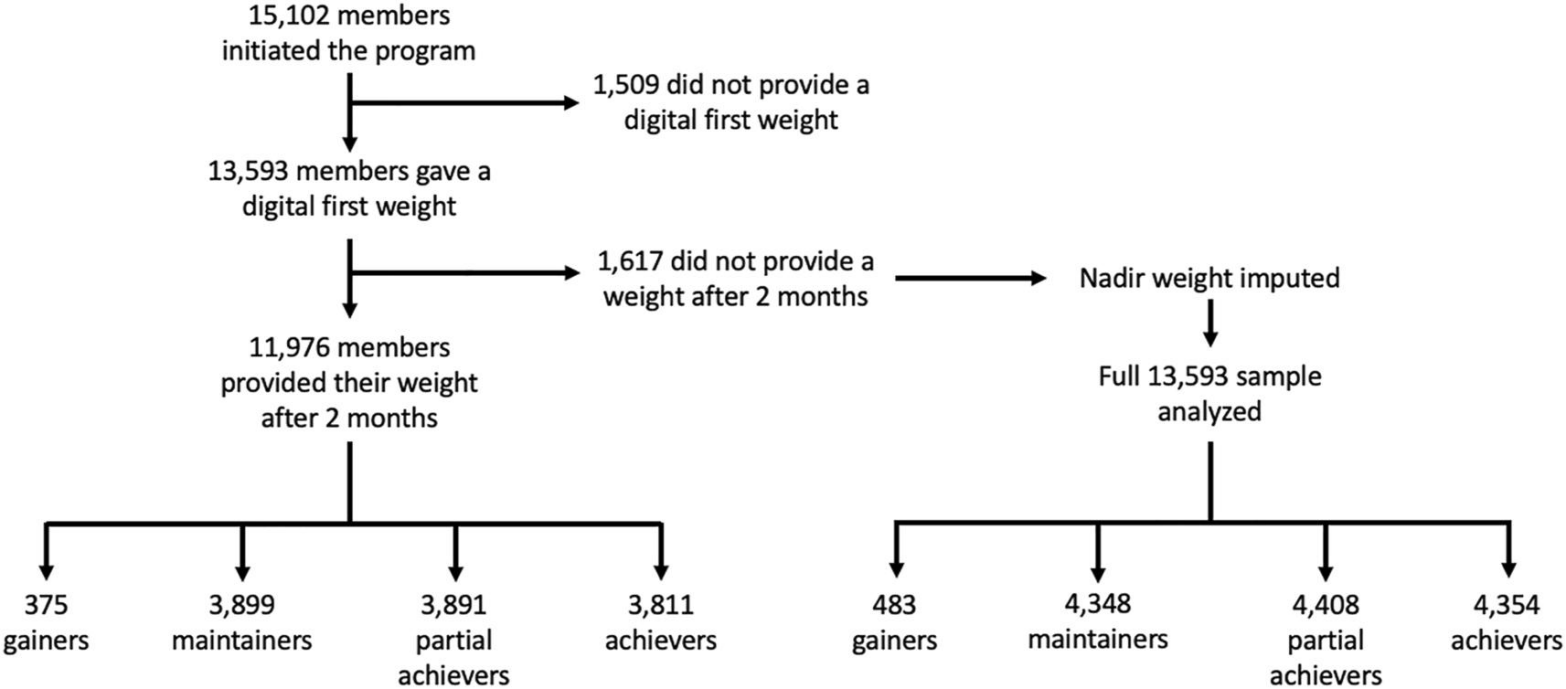
Flow of members through the study. Complete data shown on the left side of the chart. Imputed data shown on the right side.

There were 13,593 members who provided an initial digital weight and 11,976 provided one or more weights after 2 months for the nadir calculation. Of those who provided their weight, 96.9% either maintained or lost weight (Figure 1) based on the weight nadir. Table 2 shows descriptive statistics and group comparisons for all members who provided a digital first weight (*N* = 13,593).

**TABLE 2:** Demographics and baseline characteristics for complete data.

	Overall *N* = 13,593	No Nadir^0^ *n* = 1617	Gainers^1^ *n* = 375	Maintainers^2^ *n* = 3899	Partial Achievers^3^ *n* = 3891	Achievers^4^ *n* = 3811	Stats, *p*‐value
Age (years)	48.8 (0.1)	47.3 (0.3)^2,3,4^	46.0 (0.6)^2,3,4^	48.6 (0.2)^0,1,3,4^	49.6 (0.2)^0,1,2^	49.3 (0.2)^0,1,2,4^	F(413,200) = 24.8, *p* < 0.0001
Sex (% female)	79.2%	80.6%^3^	84.0%^3,4^	79.9%^3^	77.9%^0,1,2^	78.7%^1^	*χ* ^2^(8, *n* = 13,586) = 20.5, *p* < 0.01
Race (% white)	76.5%	76.7%	80.1%	75.7%	76.1%	77.3%	*χ* ^2^(4, *n* = 11,587) = 5.0, *p* = 0.29
Ethnicity (% not Hispanic)	91.9%	92.6%	93.6%	91.8%	91.3%	92.1%	*χ* ^2^(4, *n* = 11,587) = 3.7, *p* = 0.45
Initial Weight (lbs)	224.3 (0.4)	230.6 (1.3)^1,2,3,4^	221.23 (2.46)^0^	226.2 (0.8)^0,3,4^	223.1 (0.8)^0,2^	221.1 (0.8)^0,2^	F(413,588) = 13.1, *p* < 0.0001
Initial BMI (kg/m^2^)	36.5 (0.1)	37.5 (0.2)^1,2,3,4^	36.3 (0.4)^0^	36.8 (0.1)^0,3,4^	36.2 (0.1)^0,2^	36.0 (0.1)^0,2^	F(413,988) = 14.7, *p* < 0.0001
Overweight (%)	20.4%	17.2%	21.6%	19.3%	21.4%	21.6%	
Obese I (%)	28.7%	26.8%	28.8%	27.5%	29.8%	29.7%	
Obese II (%)	22.2%	22.0%	20.5%	23.0%	21.7%	22.3%	
Obese III (%)	28.7%	34.0%	28.7%	30.1%	27.1%	26.4%	
Nadir Weight (lbs)	215.0 (0.4)	N/A	228.6 (2.5)^3,4^	225.2 (0.8)^3,4^	215.6 (0.8)^1,2,4^	202.8 (0.7)^1,2,3^	F(311,972) = 157.2, *p* < 0.0001
Nadir BMI (kg/m^2^)	35.0 (0.1)	N/A	37.5 (0.4)^3,4^	36.6 (0.1)^3,4^	35.0 (0.1)^1,2,4^	33.0 (0.1)^1,2,3^	F(311,972) = 179.7, *p* < 0.0001
Prediabetes Diagnosis (% yes)	54.2%	52.5%	52.3%	52.8%^4^	54.3%	55.3%^2^	*χ* ^2^(4, *n* = 13,562) = 5.6, *p* = 0.23
Met Physical Activity Recommendations (% no at baseline)	65.8%	65.3%	69.1%^3,4^	65.0%^3^	62.5%^1,2^	63.7%^1^	*χ* ^2^(4, *n* = 13,229) = 11.3, *p* = 0.02
Initial PHQ‐2 Score	2.1 (0.0)	2.3 (0.1)^2,3,4^	2.5 (0.1)^2,3,4^	2.1 (0.0)^0,2^	2.1 (0.0)^0,1^	2.1 (0.0)^0,1^	F(413,577) = 6.9, p < 0.0001
% High Risk PHQ‐2 Score (≥3)	36.3%	40.3%	42.6%	35.6%	35.4%	35.5%	

*Note*: Superscript labels next to each weight‐loss category indicate Tukey‐corrected post hoc pairwise significant differences at *p* < 0.05. BMI = Body mass index; PHQ‐2 = Patient Health Questionnaire‐2; Met physical activity recommendations at baseline indicates performing >2 h of activity per week.

### 3.1 Weight loss

**FIGURE 2:**
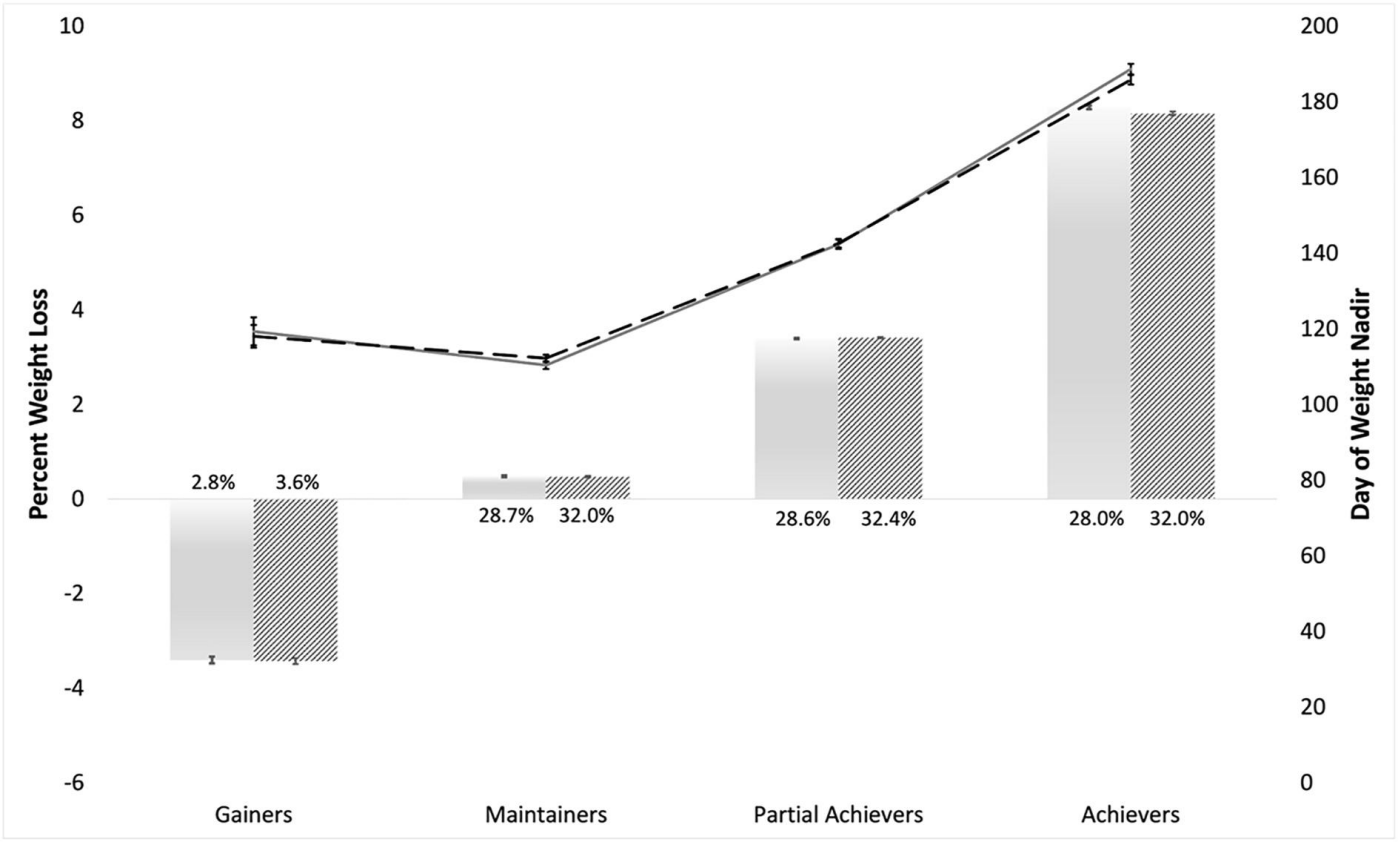
Bars: Mean (SE) percent weight loss for each weight‐loss group. Complete data shown in shaded bars and imputed data shown in dashed bars. Lines: Mean (SE) day in program of nadir weight achievement for each group. Complete data shown in solid gray line and imputed data shown in dashed black line. Percent of the overall sample (*n* = 11,976 for complete data and *n* = 13,593 for imputed data) who provided an initial weight shown above each respective bar.

There was an even distribution of members with complete weight data across the Maintenance, Partial Achievers, and Achievers groups (Figure 2). Based on the weight nadir, members who Gained weight comprised only 2.8% of the population with complete data (3.6% when considering imputed data).

**FIGURE 3:**
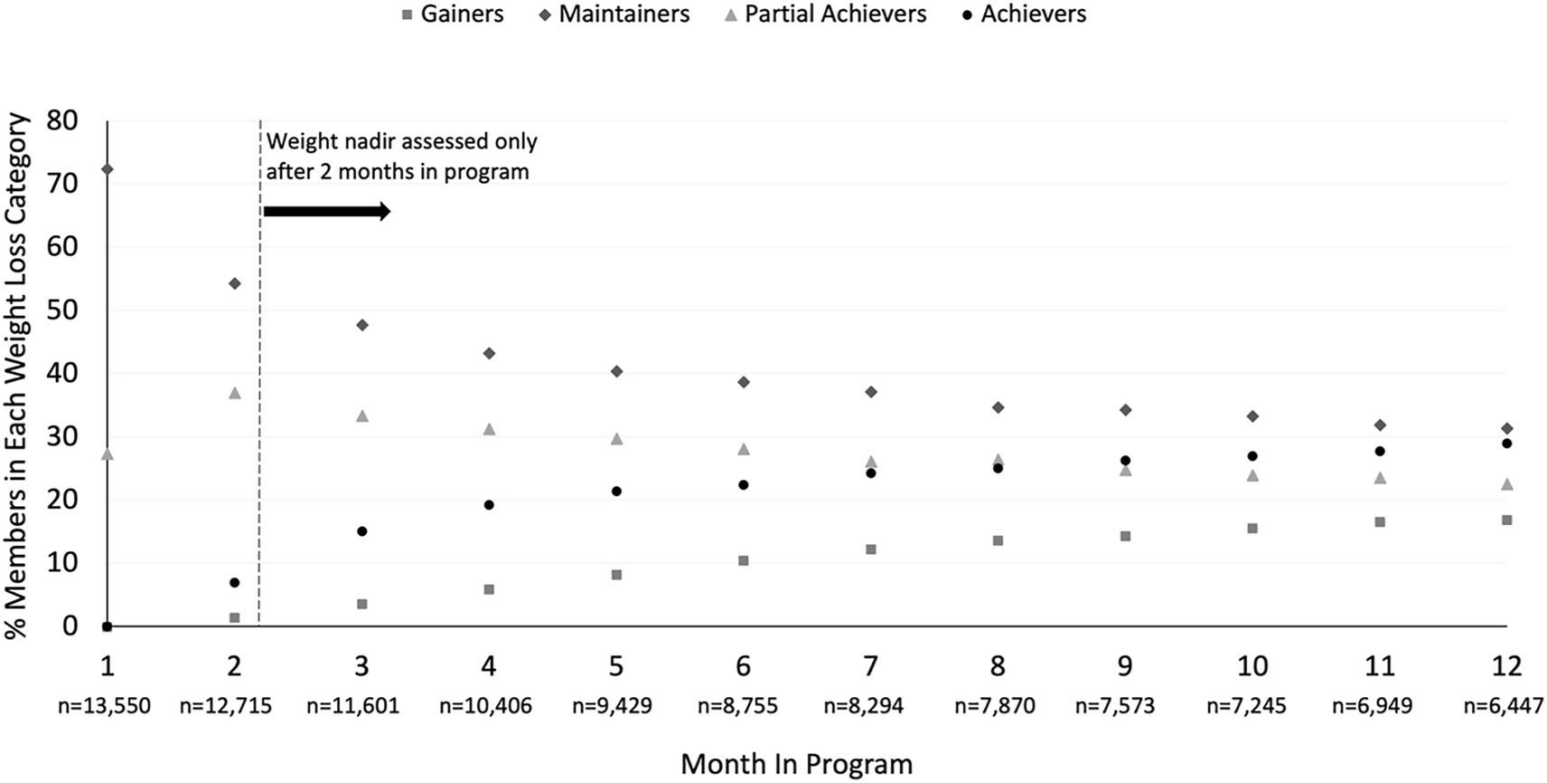
Percentage of members by weight‐loss category (Gainers, Maintainers, Partial Achievers, Achievers) per month in program. Note the expected distribution across weight‐loss categories during month 1, with most members maintaining or losing a small amount of weight at this early point in the program.

When considering the monthly proportion of members achieving each outcome across months 3 to 12 of the program (the period during which the single weight nadir occurred), an average of 23.8% of members Achieved ≥5% weight loss, 27.1% were Partial Achievers between 2% and <5% weight loss, 37.4% were Maintainers ±2%, and 11.8% Gained weight (See Figure 3).

### 3.2 Behavioral correlates of weight loss

The day in program of the achieved weight nadir increased across weight‐loss groups, with members who achieved the greatest weight loss reaching their peak the furthest into the program (Figure 2 and Table 3). The last day of active engagement in the program (i.e., any within‐app activity including logging a meal, engaging with coaching, etc.) showed a similar increasing relationship with achieved weight loss. In general, members who lost more weight exhibited greater activity within and outside of the app (e.g., tracked physical activity) (Table 3).

**TABLE 3:** Behavioral correlates of weight loss nadir.

	Overall *N* = 13,593	No Nadir^0^ *n* = 1617	Gainers^1^ *n* = 375	Maintainers^2^ *n* = 3899	Partial Achievers^3^ *n* = 3891	Achievers^4^ *n* = 3811	Stats, *p*‐value
Days until nadir (complete data)	145.9 (0.8)	N/A	119.3 (3.7)^3,4^	110.3 (1.0)^3,4^	142.3 (1.2)^1,2,4^	188.6 (1.4)^1,2,3^	F(311,972) = 711.5, *p* < 0.0001
Days until nadir (imputed data)	145.8 (0.7)	N/A	118.1 (3.0)^3,4^	112.2 (0.9)^3,4^	142.6 (1.1)^1,2,4^	185.8 (1.3)^1,2,3^	F(313,591) = 2066.2, *p* < 0.0001
Last day active engagement	216.4 (0.9)	84.0 (2.0)^1,2,3,4^	198.7 (4.9)^0,3,4^	202.2 (1.6)^0,3,4^	236.9 (1.5)^0,1,2,4^	268.0 (1.4)^0,1,2,3^	F(413,588) = 1279.0, *p* < 0.0001
Lessons completed	6.7 (0.1)	3.6 (0.1)^1,2,3,4^	4.8 (0.2)^0,2,3,4^	6.1 (0.1)^0,1,3,4^	7.2 (0.1)^0,1,2,4^	8.4 (0.1)^0,1,2,3^	F(413,588) = 289.4, *p* < 0.0001
Physical activity minutes/week	316.5 (3.3)	229.5 (7.7)^2,3,4^	221.6 (15.5)^2,3,4^	282.7 (5.2)^0,1,3,4^	327.9 (6.1)^0,1,2,4^	384.8 (7.2)^0,1,2,3^	F(413,312) = 68.0, *p* < 0.0001
Proportion hitting ≥150 min/active week in program	58.3%	45.3%^2,3,4^	46.5%^2,3,4^	56.2%^0,1,3,4^	60.1%^0,1,2,4^	64.7%^0,1,2,3^	*χ* ^2^(4, n = 13,317) = 192.3, *p* < 0.0001
Weigh‐ins/active week	1.8 (0.0)	0.9 (0.0)^2,3,4^	1.0 (0.1)^2,3,4^	1.5 (0.0)^0,1,3,4^	1.9 (0.0)^0,1,2,4^	2.5 (0.0)^0,1,2,3^	F(413,588) = 247.7, *p* < 0.0001
Meals/active week	4.9 (0.1)	5.1 (0.2)^1^	3.4 (0.2)^0,2,3,4^	4.7 (0.1)^1^	4.9 (0.1)^1^	5.0 (0.1)^1^	F(413,588) = 8.6, *p* < 0.0001
Coaching convos/active week	11.1 (0.1)	11.5 (0.2)^1,2^	8.7 (0.3)^0,2,3,4^	10.7 (0.1)^0,1,4^	11.0 (0.1)^1,2,4^	11.8 (0.1)^1,2,3^	F(413,588) = 23.3, *p* < 0.0001

*Note*: Superscript labels next to each weight‐loss category indicate Tukey‐corrected post hoc pairwise significant differences at *p* < 0.05. All engagement variables normalized to active time in the program.

### 3.3 Cost savings

**FIGURE 4:**
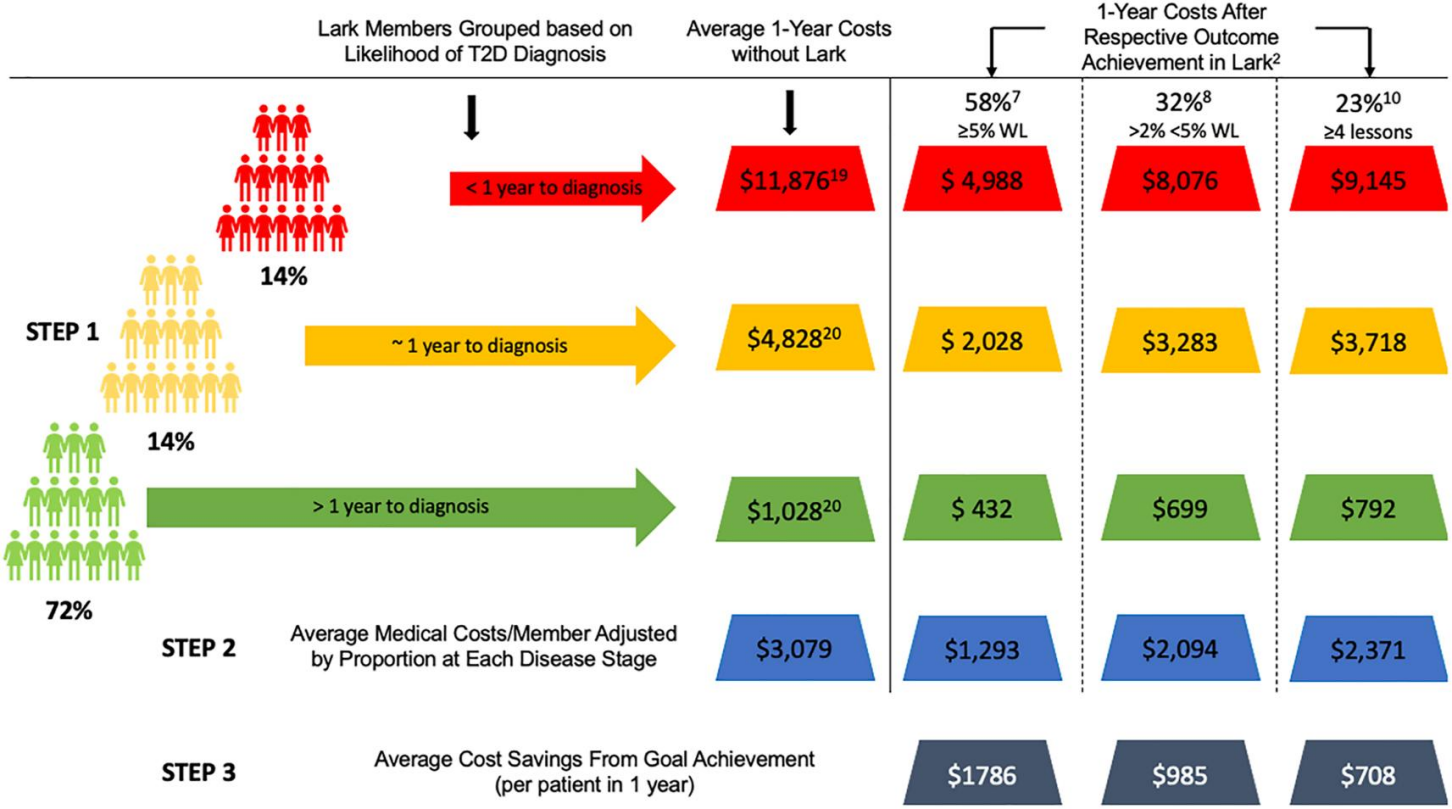
Schematic of average 1‐year costs based on the likelihood of type 2 diabetes (T2D) diagnosis (each member falls into only 1 risk category: high (red), medium (yellow), or low (green)) and the respective cost savings of the various outcome or engagement achievements due to the associated reduction in the risk of conversion to T2D (STEP 1). Each member assigned only 1 of the following: 58% reduction in risk for achieving ≥5% weight loss, 32% reduction in risk for achieving >2% but <5%, or 23% reduction in risk for completing ≥4 educational lessons. To arrive at the average medical costs adjusted by proportion at each disease stage (STEP 2), multiply the figure provided in the respective column by the proportion associated with each risk level and sum (e.g., for 58% reduction column ($4988 × 0.14 + $2028 × 0.14 + $432 × 0.72) = $1293). To arrive at the average cost savings for each goal achievement (STEP 3), subtract the average medical costs adjusted by proportion at each disease stage from the costs without Lark (e.g., $3079 –$1293 = 1786).

Based on self‐report, 3.5% of members responded affirmatively to a question asking whether they had been diagnosed with T2D during the program. This figure is consistent with the data from Craff et al.,^19^ who observed that individuals enrolled in a DPP had a ~4% annual conversion rate to T2D. To provide a conservative cost‐savings estimate for the reduction in risk of T2D, only those members with complete data received estimates of cost savings. Each of the three outcomes considered (≥5% weight loss, >2% to <5% weight loss, and ≥4 lessons) along with the estimated reduction in risk of T2D and resulting cost savings are shown in Figure 4. Each member only received acknowledgment for one achievement based on their weight nadir. Given the 3811 members who achieved ≥5% weight loss in the program, the estimated cost savings of this outcome totaled $6,806,446 ($1786 × 3811). For the 3891 members who achieved >2% but <5%, the estimated savings totaled $3,832,635 ($985 × 3891). Finally, for the 3274 members who did not achieve one of the weight loss goals, but completed ≥4 lessons, the estimated savings totaled $2,317,992 ($708  × 3274). Summing the totals for the three achievements, the estimated savings for the members followed in this study was a grand total of $12,957,073.

If based instead on the average number of members achieving each outcome across months 3–12 of the program, the cost‐savings estimate would change as follows: Given an average of 2850 members achieving ≥5% weight loss, the estimated cost savings of this outcome would total $5,090,100 ($1786 × 2850). For the 3242 members who achieved >2% but <5%, the estimated savings would total $3,193,370 ($985 × 3242). Finally, for the 4156 members who did not achieve one of the weight‐loss goals, but completed ≥4 lessons, the estimated savings totaled $2,942,448 ($708 × 4156). Summing the totals for the three achievements, the estimated savings for the members followed in this study would be a grand total of $11,229,160.

## 4 DISCUSSION

The aims of this study were to determine the proportion of members of a fully digital DPP who achieved various clinical and engagement outcomes, identify the behavioral correlates of weight loss, and estimate the potential cost savings of those achievements. Considering members who initiated the program and provided an initial digital weight, there was a retention of 88% for the calculation of weight nadir. Only ~3% of these members gained weight during the program, and the remaining members were evenly split among achieving ≥5% weight loss, losing >2% but <5%, and weight maintenance ±2%. In support of the primary study hypothesis, members who actively engaged with the program for the longest duration and exhibited the greatest in‐app activity lost the most weight. Thus, the primary findings of this paper replicate previous research demonstrating that greater and longer engagement in a DPP is associated with larger weight loss.^33^, ^34^

Observed weight loss was related to the time spent engaging in the program. Members who provided a weight nadir measurement the furthest into the program lost the most weight. Across all members, the weight nadir occurred, on average, between program day 142 (imputed data) and day 146 (complete data) (i.e., just under 5 months). The timing of this nadir is consistent with the first 6 months of the DPP being the active “weight‐loss” phase, followed by a 6‐month maintenance phase.^25^ Considering both the complete data and the imputed data for the weight nadir, ~97% of members maintained or lost weight during this active phase and ~64% lost weight. These findings are notable given that the average starting BMI of members in this study was 36.5 kg/m^2^, falling into Class II Obesity, and over a quarter were in Class III Obesity.^35^ Thus, the digital DPP is enrolling individuals in need of lifestyle modification and helping these members, who might otherwise gain ∼0.42 kg/year on average,^10^ to lose meaningful weight.

The last day of active program engagement was on average day 216 (or 31 weeks) across all members. This is comparable to a recent large study from the Diabetes Prevention Research Group that analyzed 41,000 members of in‐person DPPs and observed a median retention of 28 weeks (IQR 15, 41).^34^ However, comparing in‐person and digital DPPs may not be of high importance given that both are recognized by the CDC^36^ and perceived by participants to be similarly useful and beneficial for different reasons.^37^ Having different modes of delivery enables participating members to select a program that best meets their individual needs. The program that offers the best fit for a particular member should be the one that encourages the longest retention and thus facilitates the best outcomes.

There were significant differences in baseline demographics and characteristics between the weight‐loss groups. Members who lost weight were older than those who gained weight (49 vs. 46 years, respectively), which is also reported in aggregate data from the National DPP.^34^ Gainers were also more likely to be female, less likely to meet physical activity guidelines at baseline, and at a higher risk for depression based on their PHQ‐2 score.^28^ Interestingly, while gainers did not have a higher body weight at baseline, members who did not provide a weight for the calculation of weight nadir had a significantly higher body weight at baseline. Thus, the percent weight loss data were not missing completely at random, and accordingly, a multivariate imputation procedure estimated the missing values while accounting for the differences in baseline weight as well as other covariates.

Achievers who lost the greatest amount of weight were actively engaged with the program content and mobile application for the longest duration. They also engaged more with coaching, logged more meals, and weighed in at a greater frequency than members who lost less weight. These behaviors are consistent with previous reports of successful weight‐loss strategies.^38^, ^39^ Previous research on the Lark DPP demonstrated that members who weighed ≥2 times per week were significantly more likely to reach ≥5% weight loss.^27^ Consistent with that finding, the Achievers in this study averaged 2.5 weigh‐ins per week. Additionally, members who completed a minimum threshold of educational lessons had greater weight loss at 12 months compared to those who did not achieve this threshold.^27^ These types of engagement in digital health have also been shown to be related to clinically relevant outcomes by others.^40^, ^41^ However, members of digital health programs differ in the types of engagement they seem to prefer.^42^ For example, Hori et al.,^42^ showed that “Data‐Driven” members tended to weigh frequently and track their progress, while “Learners” conversed more with the AI‐powered coach and engaged with educational content. Thus, encouraging members to engage with application features that facilitate better outcomes, but differ from their preferences, may require customizing the ways members can engage with these features.

Most members (66% overall) reported that they did not meet physical activity recommendations at baseline, indicating that they engaged in less than 2 hours of physical activity per week. However, during the program, over half of the weight Maintainers, Partial Achievers, and Achievers engaged in ≥150 min/week of physical activity. As expected, those who engaged in the greatest amount of activity lost the most weight during the program. Regular physical activity yields many physiological benefits related to improving glycolipid metabolism.^43^ Kriska et al.,^44^ showed that physical activity participation alone reduced the incidence of T2D. Specifically, they observed a 12% reduction in risk of T2D per 6 MET‐hr. (120 min) of activity per week for participants in a lifestyle modification program, particularly those who started the program with relatively low baseline levels of physical activity. The educational content in the CDC T2 curriculum lessons, and the coaching reminders to engage in activity and suggestions on how to be active, may have helped to facilitate positive changes in members' activity levels.

The cost‐savings estimates presented in this study are consistent with healthcare cost‐savings studies in the literature from other digital health programs that had access to medical claims data.^45^ The cost‐savings estimates provided in this study drew from well‐established literature on the relative risk for incident diabetes^7^, ^30^ and the reduction in risk afforded by achieving clinical and engagement outcomes.^7^, ^8^, ^10^ Collectively, the results demonstrate the proportion of members in the program that can be expected to achieve various clinical and engagement outcomes and the financial value tied to these outcomes. The average cost of a DPP is ~$500 to $800 per member per year.^46^, ^47^ Many of these programs have high staffing costs due to employing many coaches. The advantage of a scalable program delivered by AI is that it is more affordable due to reduced staffing needs. Per member rates for an AI‐powered program typically fall ~30%–50% below market rates (including CDC estimates). Exact pricing depends on many factors such as the type of pricing model (e.g., population‐based, enrollment‐based, achievement‐based) and business‐to‐business deal considerations such as population size and characteristics, guarantees/risk, marketing privileges, levels of integration, and product customizations.

The cost‐savings estimates in this study are conservative. These estimates only used the complete data, not the imputed data, and therefore only included directly observed outcome. Based on the imputed outcomes, there were likely more members who achieved weight loss, despite not continuing to log their weights, that were not included in these estimates. Additionally, members who were Partial Achievers only received a percent risk reduction for T2D based on the low end of their weight‐loss range (2%), yet there was likely additional value for members who lost more than 2% up to <5%.

Additional areas of value not considered in this iteration of the cost‐savings model include the benefits of physical activity, the deployment of screener surveys (e.g., PHQ‐2 for depressive symptoms), escalations within the mobile application for out‐of‐range values, and reminders to complete preventive care (e.g., annual check‐ups, vaccinations). Previous research has shown that only 8% of US adults aged 35 and older had received all the recommended clinical preventive services.^48^ Implementing screenings, escalations, and reminders in a digital health app can help to enhance value‐based care by supplementing preventive care and reducing care inefficiencies, cost, and administrative burden.^49^

This study did not have a control group; however, the Lark DPP reflects the real‐world implementation of a lifestyle modification program, in which individuals self‐select whether they will participate. Without a control group, it is not possible to control for latent member characteristics such as relatively high levels of motivation that could have influenced the present findings. Despite this limitation, the National DPP is a well‐studied program with established clinical efficacy^7^ and effectiveness.^50^ The educational content used in this digital version of the program is the official, approved PreventT2 curriculum.^28^ Self‐selection into any DPP likely represents a group of individuals with higher motivation than those who do not choose to enroll in the program.

Since the multivariate imputation approach included baseline body weight, assuming a missing at random (MAR) pattern was reasonable. However, it is also important to consider reasons why the data may not have been MAR, and thus be non‐ignorable. For example, an inspection of each member's full available weight trajectory could reveal that individuals with increasing weight subsequently dropped out. However, given that the proportion of members who gained weight also increased slightly over the 12‐month intervention period, it is unlikely that weight gain is the only reason for attrition. Weight trajectories will be a focus of future studies to identify any such patterns.

Medical claims data were not available for a direct economic evaluation. Therefore, the cost‐savings model presented here demonstrates the potential for cost savings based on National DPP literature. This type of approach is an important step toward conceptualizing program value and is of particular interest to payers offering this program as a covered service. Additionally, a literature‐based model could be viewed as preferable in the context of this presentation since it provides estimates using larger sample sizes than most claims model economic evaluations, allowing for better generalization.

## 5 CONCLUSIONS

Members of a fully digital DPP powered by AI achieved clinical and engagement outcomes during their participation in the program that confer important health benefits and resulted in potential cost savings. Members who exhibited greater engagement in healthy lifestyle behaviors during the program including weighing and meal logging, conversing with the AI coach, and being physically active lost the most weight. A conservative, literature‐based, cost‐savings model demonstrated the potential financial value associated with achieving clinical and engagement outcomes. As research reveals tangible value for other aspects of digital health programs such as the delivery of health screenings, escalations, and reminders, the cost‐savings potential of these programs may be fully realized.

## AUTHOR CONTRIBUTION

All authors contributed equally to this manuscript.

## CONFLICT OF INTEREST

OHB, MR, LAG, KGL, and SAG are employed by Lark.
